# Systemic administration of IGF-I enhances healing in collagenous extracellular matrices: evaluation of loaded and unloaded ligaments

**DOI:** 10.1186/1472-6793-7-2

**Published:** 2007-03-26

**Authors:** Paolo P Provenzano, Adriana L Alejandro-Osorio, Kelley W Grorud, Daniel A Martinez, Arthur C Vailas, Richard E Grindeland, Ray Vanderby

**Affiliations:** 1 Dept. of Biomedical Engineering, University of Wisconsin, Madison, WI, USA; 2 Dept. of Biomolecular Chemistry, University of Wisconsin, Madison, WI, USA; 3 Dept. of Orthopedics and Rehabilitation, University of Wisconsin, Madison, WI, USA; 4 Dept. Health and Human Performance, University of Houston, Houston, TX, USA; 5 Dept. of Mechanical Engineering and The Biomedical Engineering Program, University of Houston, Houston, TX, USA; 6 Life Sciences Research Division, NASA-Ames Research Center, Moffett Field, CA, USA

## Abstract

**Background:**

Insulin-like growth factor-I (IGF-I) plays a crucial role in wound healing and tissue repair. We tested the hypotheses that systemic administration of IGF-I, or growth hormone (GH), or both (GH+IGF-I) would improve healing in collagenous connective tissue, such as ligament. These hypotheses were examined in rats that were allowed unrestricted activity after injury and in animals that were subjected to hindlimb disuse. Male rats were assigned to three groups: ambulatory sham-control, ambulatory-healing, and hindlimb unloaded-healing. Ambulatory and hindlimb unloaded animals underwent surgical disruption of their knee medial collateral ligaments (MCLs), while sham surgeries were performed on control animals. Healing animals subcutaneously received systemic doses of either saline, GH, IGF-I, or GH+IGF-I. After 3 weeks, mechanical properties, cell and matrix morphology, and biochemical composition were examined in control and healing ligaments.

**Results:**

Tissues from ambulatory animals receiving only saline had significantly greater strength than tissue from saline receiving hindlimb unloaded animals. Addition of IGF-I significantly improved maximum force and ultimate stress in tissues from both ambulatory and hindlimb unloaded animals with significant increases in matrix organization and type-I collagen expression. Addition of GH alone did not have a significant effect on either group, while addition of GH+IGF-I significantly improved force, stress, and modulus values in MCLs from hindlimb unloaded animals. Force, stress, and modulus values in tissues from hindlimb unloaded animals receiving IGF-I or GH+IGF-I exceeded (or were equivalent to) values in tissues from ambulatory animals receiving only saline with greatly improved structural organization and significantly increased type-I collagen expression. Furthermore, levels of IGF-receptor were significantly increased in tissues from hindlimb unloaded animals treated with IGF-I.

**Conclusion:**

These results support two of our hypotheses that systemic administration of IGF-I or GH+IGF-I improve healing in collagenous tissue. Systemic administration of IGF-I improves healing in collagenous extracellular matrices from loaded and unloaded tissues. Growth hormone alone did not result in any significant improvement contrary to our hypothesis, while GH + IGF-I produced remarkable improvement in hindlimb unloaded animals.

## Background

Insulin-like growth factor-I (IGF-I) plays a crucial role in muscle regeneration, can reduce age-related loss of muscle function, and cause muscle hypertrophy when over-expressed [1-5]. These effects appear to be largely mediated by promoting proliferation and differentiation of satellite cells [3] as well as promoting recruitment of proliferating bone marrow stem cells to regions of muscle tissue damage [6]. Furthermore IGF-I and growth hormone (GH) are involved in a large variety of physiologic functions and are reported to promote healing and repair in bone [7,8], cartilage [9-11], gastric ulcers [12], muscle [13,14], skin [15-17], and tendon [18,19]. This action is largely mediated by the fact that GH and IGF-I directly affect cells involved in the healing response [20-30], with IGF-I having endocrine action, as well as local expression, resulting in autocrine and/or paracrine signaling that plays a role in proliferation, apoptosis, cellular differentiation, and cell migration [31-36]. Insulin-like growth factor-I also stimulates fibroblast synthesis of extracellular matrix (ECM) molecules such as proteoglycans and type I collagen [18,30,37,38], and IGF-I mRNA and protein levels are increased in healing ligaments [39] and tendons [40], respectively. As such, IGF-I is of particular interest in tissue regeneration due to its influence on cell behavior and role in type I collagen expression.

Fibrous connective tissues, such as ligament and tendon, are composed primarily of type I collagen with type III collagen levels increased during healing [41]. During development, collagen molecules organize into immature collagen fibrils that fuse to form longer fibrils [42-45]. In mature tendon and ligament these fibrils appear to be continuous and transfer force directly through the matrix [46]. In ligament, groups of fibrils form fibers and it is these fiber bundles that form fascicles; the primary structural component of the tissue. Previous studies in healing ligament have shown that disruption of the medial collateral ligament (MCL) results in substantial reduction in mechanical properties which does not return to normal after long periods of healing [47]. Such tissue behavior is likely associated with matrix flaws, reduced microstructural organization, and small diameter collagen fibrils in the scar region of the ECM [48-50]. Additionally, during normal ligament healing collagen fibrils from residual tissue fuse with collagen fibrils formed in the scar region [51]. However, in tissues which are exposed to a reduced stress environment such as joint immobilization [52] or hindlimb unloading [48] collagen fibers contain discontinuities and voids [48] which likely account for the substantial decrease in tissue strength when compared to ligaments experiencing physiologic stress during healing. Since soft tissue injuries are common and do not heal properly in a stress-reduced environment [48,52], such as is present during prolonged bed rest or spaceflight, methods to further understand tissue healing and promote tissue healing require study.

The purpose of this study is to test the hypotheses that systemic administration of IGF-I, GH, or GH+IGF-I will improve healing in a collagenous ECM. Furthermore, since the addition of GH has been shown to up-regulate IGF-I receptor [53], levels of IGF-I receptor in healing tissues were examined in order to begin to elucidate the molecular mechanism by which GH and/or IGF-I may be acting to locally to stimulate tissue repair. Since IGF-I and GH are feasible for clinical use, identifying benefits from short-term systemic administration, such as improved connective tissue healing, have great potential to improve the human condition. The hypotheses are examined in animals that are allowed normal ambulation after injury and in animals that are subjected to disuse through hindlimb unloading. The MCL was chosen as a model system since this ligament, unlike tendons, has no muscular attachment and therefore possible alterations in muscle strength after IGF-I and/or GH treatment do not impose substantial differential loads on the ligament during hindlimb unloading. Furthermore, since MCLs have two attachments/insertions into bone, and hindlimb unloading/disuse is known to reduce the mechanical properties of bone [48,54], failure location was recorded for all mechanical testing. Results indicate improved mechanical properties and collagen organization and composition of the collagenous extracellular matrix following treatment with IGF-I in both ambulatory and hindlimb unloaded animals or IGF-I+GH in hindlimb unloaded animals.

## Results

Initial body weights were not different between groups and no wound infections or other apparent complications associated with surgery were observed. All of the ambulatory animals returned to normal cage activity shortly after surgery, and no treatment complications were observed in the suspended animals. The hindlimb unloaded (HU) animals were not able to gain weight as rapidly as the ambulatory animals, thus significant differences (p < 0.0001) in body weight were observed between groups after 10 days of healing. The GH, IGF-I, and GH+IGF-I treatment increased body weight in HU animals (compared to HU animals receiving only saline) but this difference was not statistically significant (p = 0.49, 0.49, and 0.26, respectively). No significant differences in body weight were present between the ambulatory animals (all p values > 0.38). At tissue harvest all healing ligaments showed a bridging of the injury gap with translucent scar tissue. In the ambulatory animals receiving GH, three animals had hematomas and tissue adhesions in the surgical site. In all other animals no gross differences in the tissues or the surgical wound site were observed.

The location of structural failure in each ligament was examined, revealing that the majority of the femur-MCL-tibia complexes failed in the MCL proper, not at the ligament to bone insertion sites, nor by bone avulsion (Table 1). Sham control ligaments failed in the tibial third of the ligament during 100% of the tests. For all other groups, the primary location of failure was the scar region of the ligament. The only exceptions to this were one failure in the tibial third of the ligament in the Amb + IGF group, one tibial avulsion in the HU + Sal group, and one tibial avulsion in the HU + IGF group, indicating that a significant portion of the failure locations was in the scar region (all p values < 0.01). These results indicate the dominant effects in the measured mechanical properties are due to changes in ligamentous tissue and not in the insertions.

**Table 1:** Failure location of the bone-ligament-bone complex at 3 weeks post-injury.

***Failure Location***	**Scar Region**	**Tibial Third of the Ligament**	**Tibial Avulsion**
**Sham Control**	N/A	6	0
**Amb + Saline**	6	0	0
**Amb + GH**	6	0	0
**Amb + IGF**	5	1	0
**Amb + GH + IGF**	6	0	0
**HU + Saline**	5	0	1
**HU+ GH**	6	0	0
**HU + IGF**	5	0	1
**HU+ GH + IGF**	6	0	0

The strong majority (>96%) of tissues failed in the ligament and not by avulsion, indicating that the measured mechanical changes are due to changes in the ligament properties and not bone.

**Figure 1:**
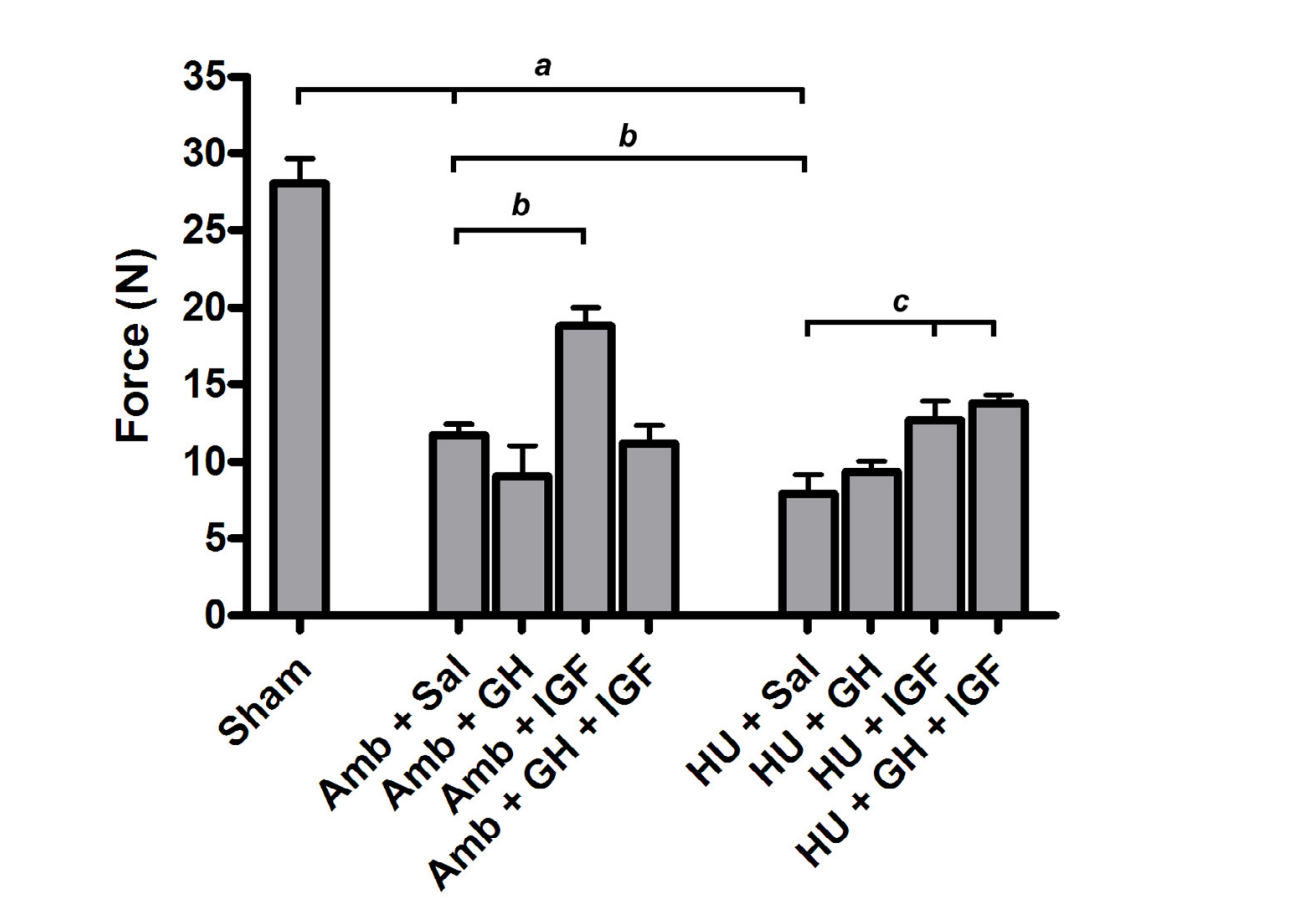
Maximum force values at 3 weeks (mean ± S.E.M.). Maximum force was significantly decreased in tissues from hindlimb unloaded (HU) animals receiving saline when compared to tissues from Amb + Sal animals (p = 0.03). The addition of IGF-I significantly improved maximum force in tissues from ambulatory healing animals when compared to tissues from ambulatory healing animals that received saline (p = 0.0002). Addition of IGF-I and GH+IGF-I in HU animals significantly increased maximum force values when compared to hindlimb unloaded animals receiving saline (p = 0.0074 and p = 0.0013, respectively). *a *significantly different from Sham group; *b *significantly different from Amb + Sal group; *c *significantly different from HU + Sal group.

**Figure 2:**
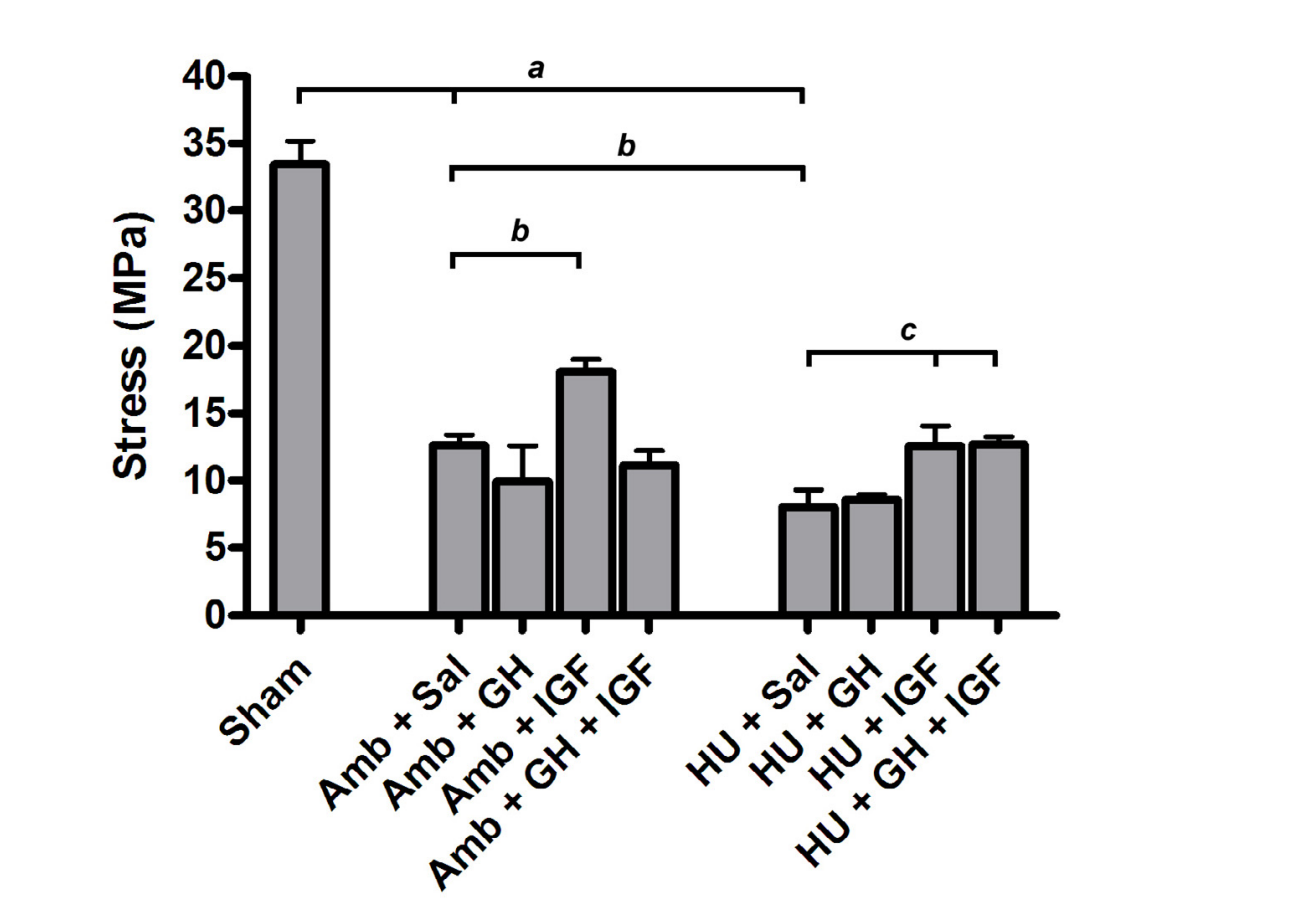
Ultimate stress values at 3 weeks (mean ± S.E.M.). Ultimate stress was significantly decreased in tissues from unloaded animals receiving saline when compared to tissues from Amb + Sal (p = 0.022). The additional of IGF-I significantly improved ultimate stress levels in tissues from ambulatory healing animals when compared to tissues from ambulatory healing animals which received saline (p = 0.0077). Addition of IGF-I and GH+IGF-I in hindlimb unloaded animals significantly increased ultimate stress values when compared to hindlimb unloaded animals receiving saline (p = 0.0236 and p = 0.0202, respectively). *a *significantly different from Sham group; *b *significantly different from Amb + Sal group; *c *significantly different from HU + Sal group.

**Figure 3:**
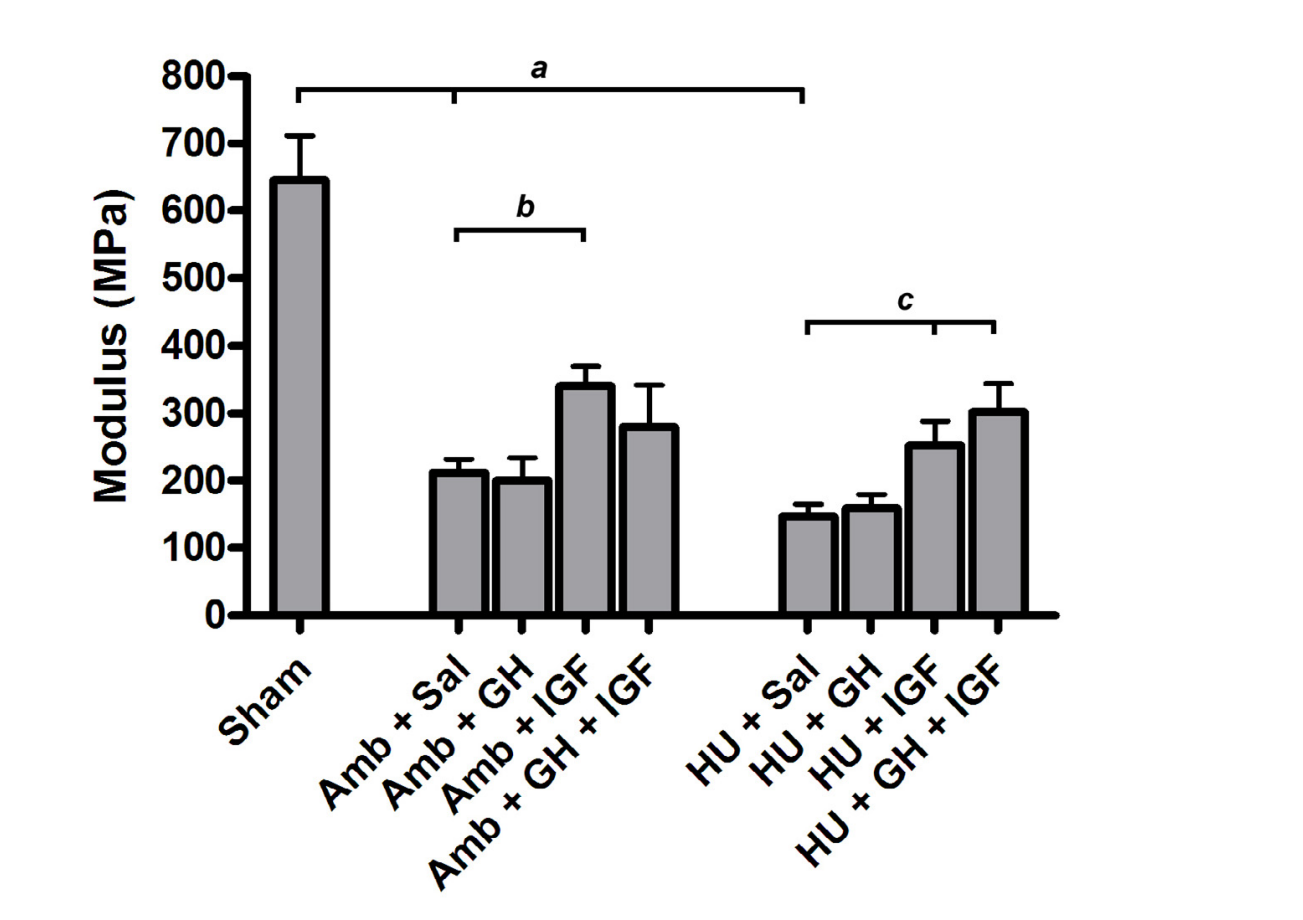
Elastic modulus values at 3 weeks (mean ± S.E.M.). The additional of IGF-I significantly improved elastic modulus levels in tissues from ambulatory healing animals when compared to tissues from ambulatory healing animals which received saline (p = 0.049). A significant increase in elastic modulus was present in tissues from hindlimb unloaded animals after the addition of IGF-I or GH+IGF-I when compared to hindlimb unloaded animals receiving saline (p = 0.049 and p = 0.014, respectively). *a *significantly different from Sham group; *b *significantly different from Amb + Sal group; *c *significantly different from HU + Sal group.

Substantial differences in tissue mechanical properties were present between groups. Hindlimb unloading adversely affected ligament healing while IGF-I or GH+IGF-I had a substantial effect on ligament healing in either ambulatory or hindlimb unloaded animals. Maximum force (Fig. 1) was significantly different between tissues from Sham and both Amb + Sal and HU + Sal groups (p = 0.0001 and p = 0.0001, respectively). Hindlimb unloaded animals had significantly decreased maximum force in the MCLs when compared to ambulatory healing animals (Amb + Sal; p = 0.03). In ambulatory animals the addition of IGF-I significantly improved maximum force values by approximately 60% when compared to ambulatory healing animals that received saline (p = 0.0002). Addition of IGF-I and GH+IGF-I in hindlimb unloaded animals significantly increased maximum force values (60% and 74%, respectively) when compared to HU + Sal animals (p = 0.0074 and p = 0.0013, respectively). In fact, addition of IGF-I or GH+IGF-I to hindlimb unloaded animals increased force to be comparable with Amb + Sal animals. Growth hormone alone did not have a significant effect within either the ambulatory or unloaded groups. However, in the unloaded group, GH increased maximum force and brought it closer to values in the Amb + Sal group, yet this increase was not statistically significant (p = 0.16). Ultimate stress (Fig. 2) was significantly decreased in tissues from both ambulatory healing and hindlimb unloaded healing animals when compared to sham control tissues (p = 0.0001 and p = 0.0001, respectively). Ligaments from HU + Sal animals had ultimate stress values that were significantly lower than saline receiving ambulatory animals (p = 0.022). Addition of only IGF-I to ambulatory animals significantly increased ultimate stress when compared to Amb + Sal animals (p = 0.0077). Delivery of IGF-I and GH+IGF-I significantly increased ultimate stress in tissues from the hindlimb unloaded animals when compared to hindlimb unloaded (plus saline) animals (p = 0.0236 and p = 0.0202, respectively). In fact, ultimate stress values after the addition of IGF-I or GH+IGF-I to hindlimb unloaded animals was comparable to ultimate stress values in ambulatory animals receiving saline. In ambulatory animals, no statistically significant effect on ultimate stress values were seen after GH or GH+IGF-I was administered. No significant differences in strain at failure were present between groups. Elastic modulus (Fig. 3) was statistically different between IGF-I and saline treated ambulatory healing animals. Addition of IGF-I in ambulatory animals resulted in an elastic modulus that had an ~49% greater mean value (p = 0.049). In hindlimb unloaded animals the addition of IGF-I or GH+IGF-I resulted in a significant increase in modulus when compared to unloaded animals receiving saline (p = 0.049 and p = 0.014, respectively). Growth hormone alone had no significant effect on elastic modulus in either group.

**Figure 4:**
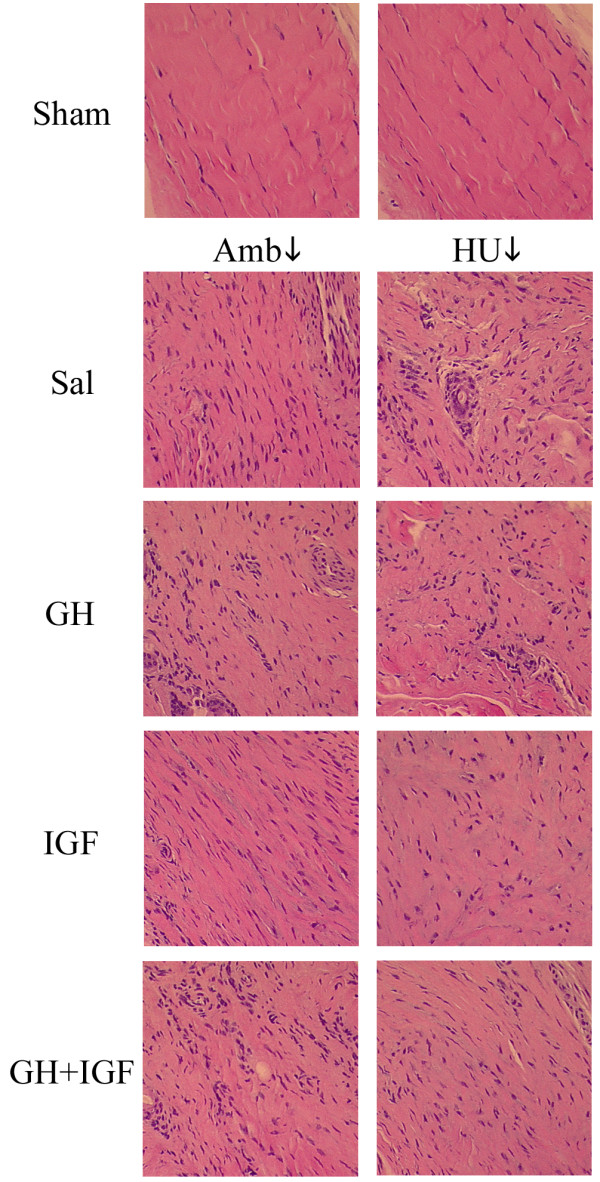
Representative tissue sections taken from the midsubstance region of control normal tissue (*Sham*) and scar tissues (*Saline, GH, IGF-I, GH+IGF-I*). The longitudinal axis of the ligament is from the *top left *to *bottom right *of each image (200X). Tissues from sham control (Sham) animals have the characteristic crimp pattern and aligned fibroblasts associated with normal tissue. Examination of scar tissue from ambulatory healing animals (*Amb + Sal*) revealed typical scar morphology with matrix disorganization and hypercellularity. In contrast to ambulatory animals, hindlimb unloaded (*HU + Sal*) animals showed abnormal scar formation with pockets of cell clusters and misaligned collagen fibers creating defects and voids by not connecting. Examination of extracellular matrices from GH treated animals revealed no improvement in matrix organization in tissues from ambulatory (*Amb + GH) *or hindlimb unloaded (*HU + GH*) animals. However, animals treated with IGF-I showed extensively increased matrix density and substantially improved matrix alignment in tissues from ambulatory (*Amb + IGF*) and hindlimb unloaded (*HU + IGF*) animals; indicating a considerable improvement in the matrix structure in animals treated with IGF-I. Animals that were treated with both GH and IGF-I showed similar improvement to that seen with IGF-I in unloaded tissues (*HU + GH + IGF*) but showed no improvement in ambulatory tissues (*Amb + GH + IGF*).

Representative H&E stained sections (Fig. 4) revealed hypercellularity and extracellular matrix disorganization in injured tissues from both ambulatory and suspended animals. Ligaments from Sham animals demonstrated the characteristic crimp pattern and aligned fibroblasts associated with normal ligament (Fig. 4). However, while Amb + Sal animals revealed normal scar morphology, tissues from HU + Sal animals had pockets of cell clusters, cellular misalignment, and misaligned collagen bundles oriented in separate directions creating discontinuities and voids within the matrix confirming a previous report [48]. Furthermore, while matrices from animals treated with GH had abnormal cell clusters and no gross improvement in matrix organization in both ambulatory and unloaded tissues, tissues from animals treated with IGF-I showed substantially increased matrix density and collagen alignment (Fig. 4); a morphology also present in tissues from HU + GH + IGF-I animals.

**Figure 5:**
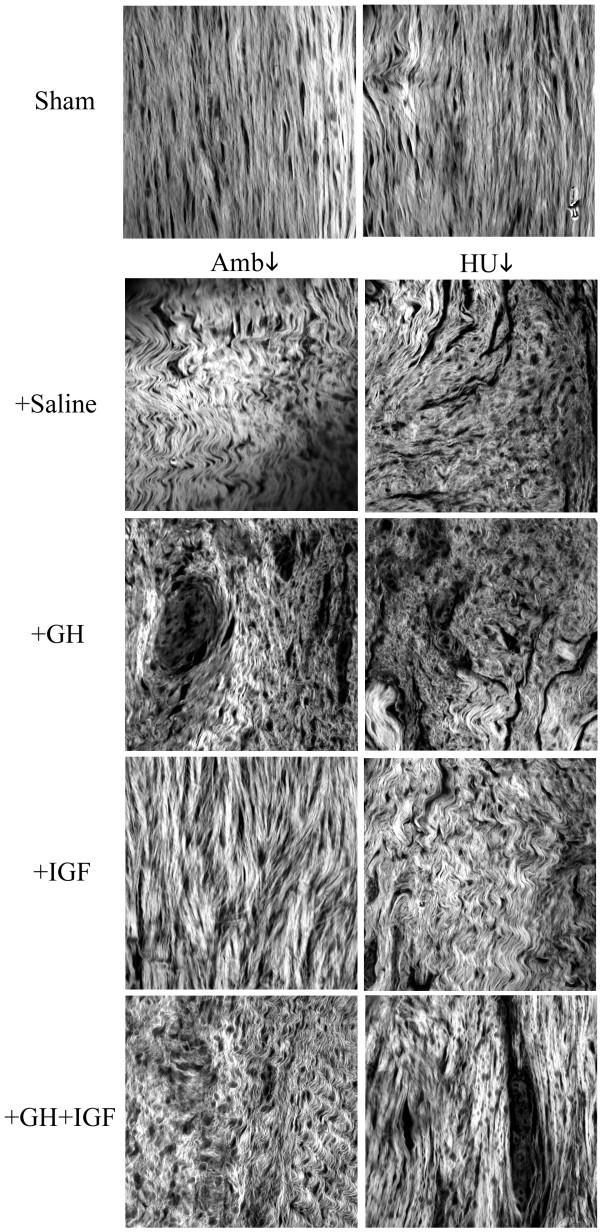
Multiphoton Laser Scanning Microscopy (MPLSM) was performed on hematoxylin and eosin sections in order to evaluate the organization and structure of the collagen matrix in more detail then allowed by conventional brightfield light microscopy. The longitudinal axis of the ligament is from the *top *to *bottom *of each image. Tissues from sham control animals have the characteristic crimp pattern associated with normal tissue (*Sham*). Scar tissue from ambulatory healing animals (*Amb + Sal*) revealed typical scar morphology with matrix disorganization while hindlimb unloaded animals (*HU + Sal*) showed good fiber aggregation but possessed abnormal scar formation with misaligned collagen fibers not directed along the longitudinal axis of the tissue creating voids and defects by not connecting. Assessment of collagen matrices from GH treated animals revealed no improvement in matrix organization in tissues from ambulatory (*Amb + GH) *or hindlimb unloaded (*HU + GH*) animals. Examination of collagen structure in animals treated with IGF-I supported data shown in Fig. 4 revealing greatly increased matrix density and considerably improved matrix alignment in tissues from ambulatory (*Amb + IGF*) and hindlimb unloaded (*HU + IGF*) animals. Animals treated with GH+IGF showed no significant improvement in ambulatory tissues (*Amb + GH + IGF*) but unloaded tissues (*HU + GH + IGF*) showed substantially increased matrix density and alignment.

**Figure 6:**
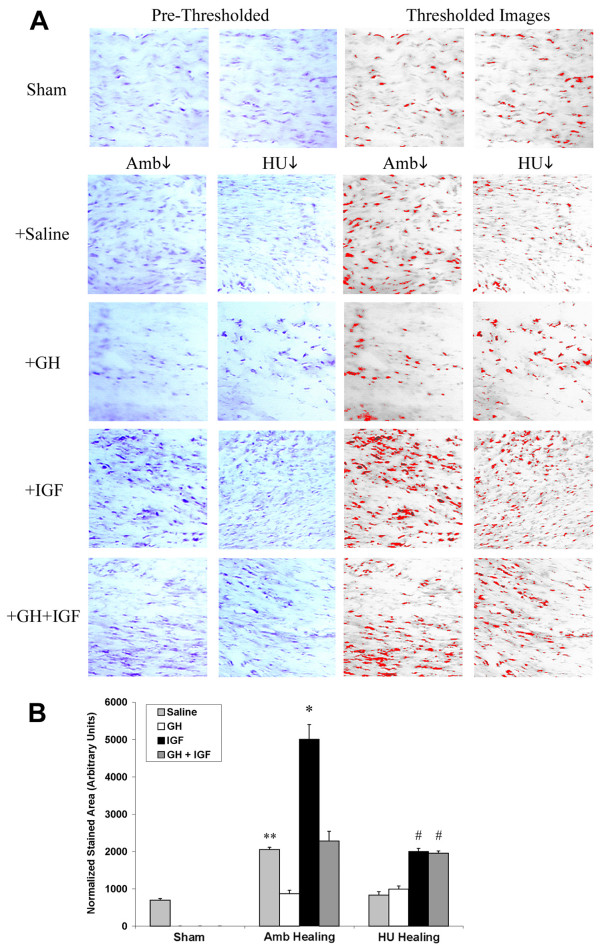
Significantly increased expression of type-I collagen in tissues from animals treated with IGF-I. (*A: Left*) Immunohistochemistry shows increased staining for type I collagen in ambulatory tissues when compared to Sham, while hindlimb unloaded tissues show no increase. In tissues from ambulatory and hindlimb unloaded animals treated with IGF-I type I collagen staining is increased. GH+IGF-I also results in increased collagen expression in unloaded tissues. (*A: Right*) Thresholded images utilized for quantitative analysis of expression. (*B*) Quantitative analysis of type I collagen expression. Ambulatory healing (Amb + Sal) tissue had significantly more collagen expression when compared to sham tissues (** p = 0.0002) while unloaded tissues did not. GH showed no significant increase between saline treated animals. Ambulatory IGF-I treated animals had significantly (*) increased type-I collagen protein expression (p = 0.0018) when compared to ambulatory animals treated with saline. Tissues from GH+IGF treated ambulatory and hindlimb unloaded animals had significantly (# p = 0.0006 and p = 0.0005, respectively) increased collagen expression when compared to hindlimb unloaded tissues given saline.

**Figure 7:**
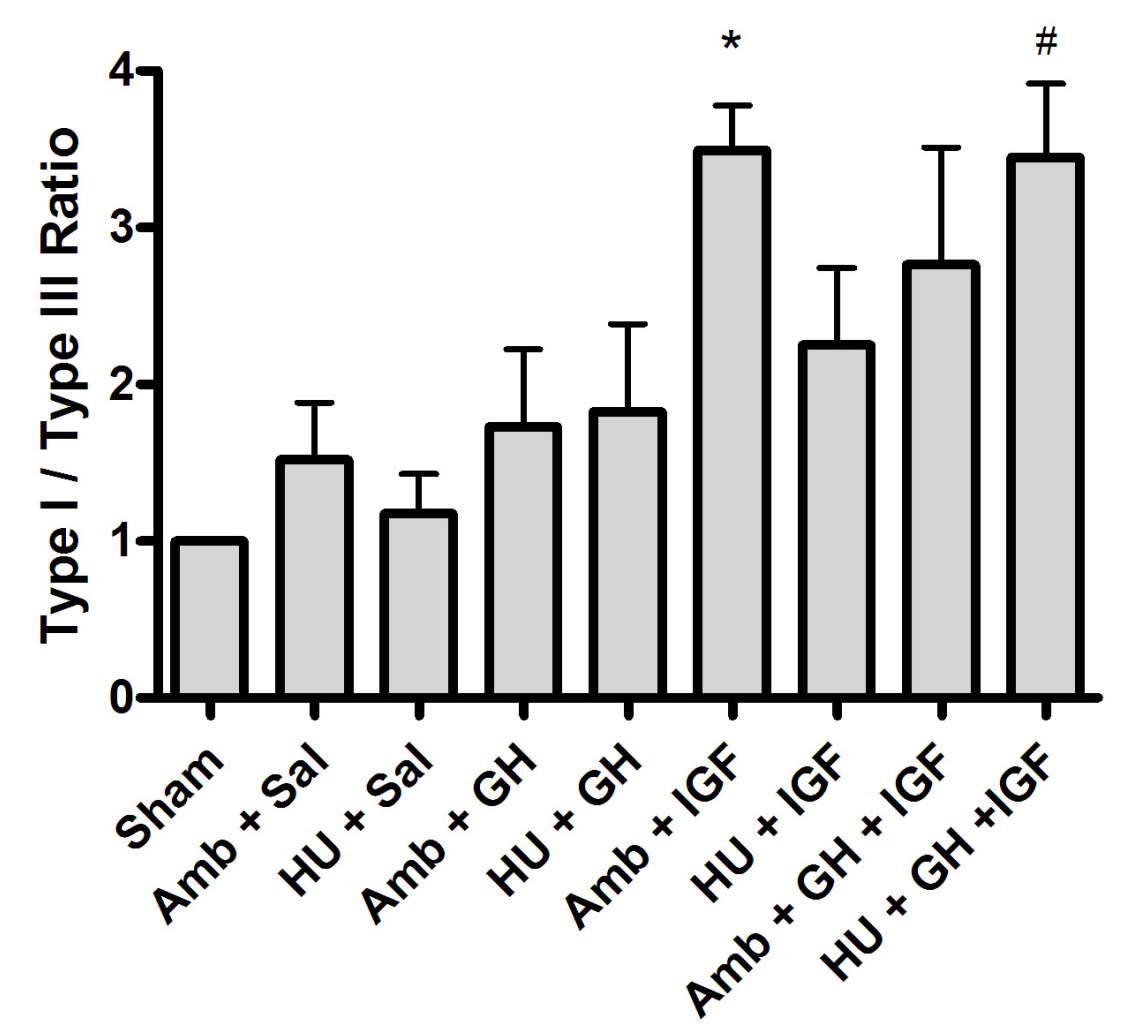
Densitometry analysis of type I and III collagen expression. Quantification of Western blots for type I and III collagen indicated that the ratio of type I to type III collagen was significantly increased in tissues from ambulatory animals treated with IGF-I (* p = 0.0129) and in unloaded tissues from GH+IGF-I treated animals (p = 0.0131), when compared to saline treated ambulatory and hindlimb unloaded tissues. Note: the values for each group were normalized to the Sham group (ratio set to one) to allow comparison of data across multiple Western blot experiments.

To further examine extracellular matrix organization in histology sections, multiphoton laser scanning microscopy (MPLSM), which allows greatly enhanced imaging of collagen structure, was employed (Fig. 5). Examination of sham, ambulatory, and hindlimb unloaded tissues supports morphological information obtained with classical histology (Fig. 4) and scanning electron microscopy [48], with tissues from HU animals showing good fiber bundle formation but fiber misalignment creating matrix discontinuities and voids (Fig. 5). Confirming results obtained from viewing H&E section with light microscopy, MPLSM analysis showed improved matrix organization in tissues from Amb + IGF-I, HU + IGF, and HU + GH + IGF-I animals (Fig. 5). In accordance with data showing increased matrix deposition and organization (Figs. 4 and 5), expression of type-I collagen was increased in tissues from IGF-I treated ambulatory (p = 0.0018) and unloaded (p = 0.0006) animals and GH+IGF-I treated unloaded tissue (p = 0.0005; Figs. 6A and 6B). Densitometry analysis further confirmed an increase in type-I collagen since the ratio of type-I to type-III collagen was significantly increased in tissues from ambulatory animals treated with IGF-I (p = 0.0129) and in unloaded tissues from GH+IGF-I treated animals (p = 0.0131), with a trend of increased levels in ambulatory GH+IGF and HU + IGF tissues (Fig. 7). Since normal ligaments are primarily composed of type I collagen and the scar region of normal healing ligaments contain an increase in type III collagen [41] that is remodeled to transition to more type I collagen rich region over the healing period, changes in the type I to III ratio are a measure of healing and may indicate part of the structural mechanism resulting in the observed differences in mechanical properties following treatment.

**Figure 8:**
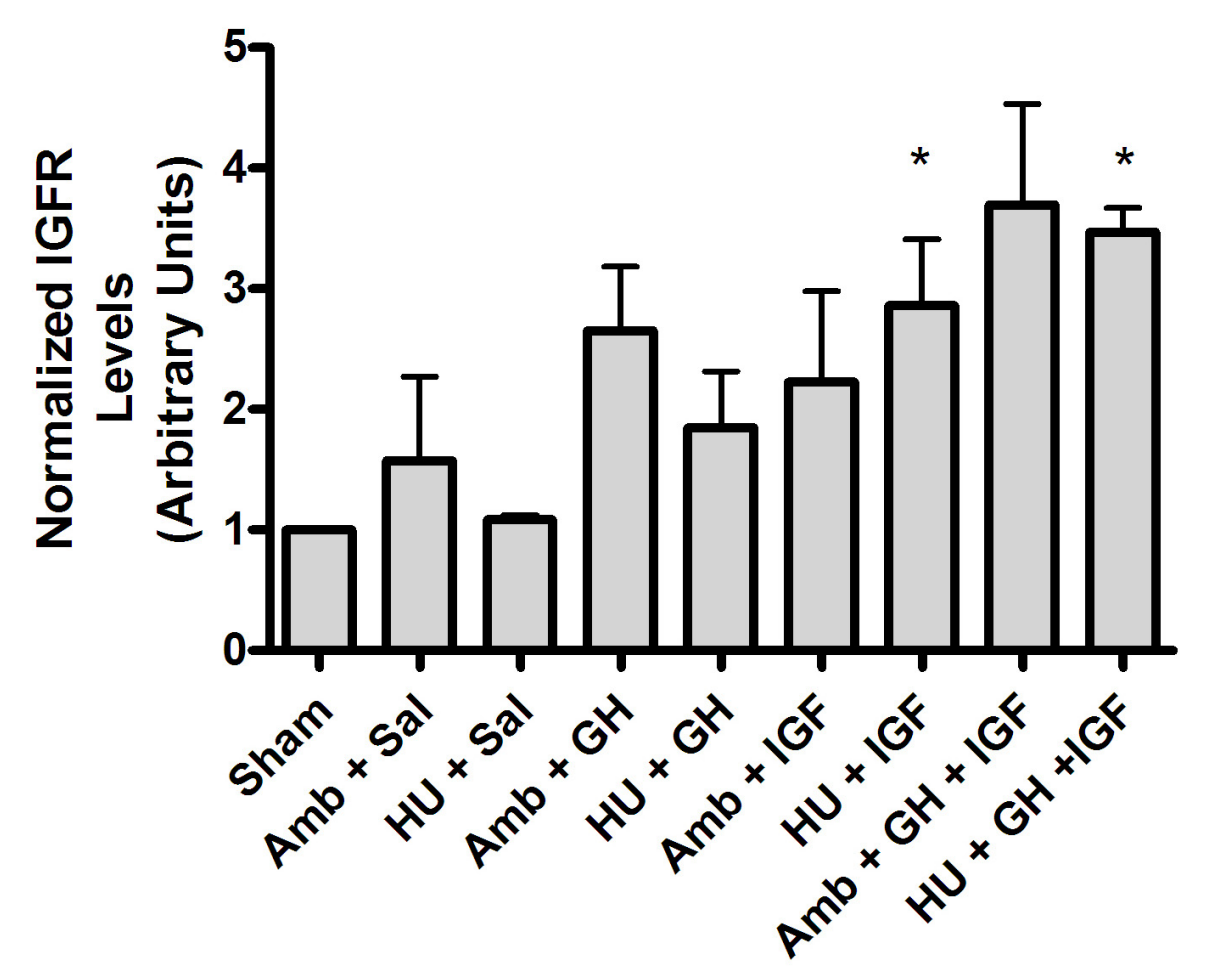
Densitometry analysis of IGF-I receptor expression. Ambulatory IGF-I and GH+IGF-I treated animals showed a strong trend of increased IGF-I receptor expression, however, this trend was not significant. In hindlimb unloaded animals treated with IGF-I or GH+IGF-I, IGF-I receptor expression was significantly increased compared to saline treated hindlimb unloaded animals, indicating a possible role for localized increases in IGF-I receptor to play a role in mediating improvements seen in the structure and function of treated ligaments.

Lastly, GH has been reported to increase levels of IGF receptors [53]. Herein, ambulatory IGF-I and GH+IGF-I treated animals showed a strong trend of increased IGF-I receptor expression, although this trend was not significant (Fig. 8). However, in hindlimb unloaded animals treated with IGF-I or GH+IGF-I, IGF-I receptor expression was significantly increased (Fig. 8). Hence, increased levels of IGF-I receptor in healing tissues may be part of the molecular mechanism by which systemic administration of IGF-I and GH+IHG-I act to locally to stimulate tissue repair.

## Discussion

Previous work in our laboratory has shown that mechanical properties and matrix organization of MCLs are substantially reduced after injury and that this impairment is significantly compounded by stress reduction through hindlimb unloading [48]. This result is confirmed by examination of the mechanical properties in MCLs from the saline receiving control groups in this study (Sham, Amb + Sal, HU + Sal) which are not significantly different from values obtained in our previous work in tissues from sham, ambulatory healing, and hindlimb unloaded animals after three weeks of healing [48]. Furthermore, structural analysis of the matrix with MPLSM confirmed previous morphological information obtained with electron microscopy [48], elucidating changes in the structure-function relationship, such as collagen fiber misalignment and matrix voids, that help explain the reduced mechanical properties associated with tissue unloading (i.e. disuse).

Results of this study demonstrate a substantial increase in healing with the systemic application of IGF-I in ambulatory and hindlimb unloaded animals and with GH + IGF-I in hindlimb unloaded animals. Since the strong majority of tissues failed in the ligament and not by avulsion, it is clear that the mechanical properties of the ligament are being evaluated and that systemic treatment with IGF-I or GH+IGF-I improves the integrity of the collagenous matrix. This finding is in contrast with results from healing tendons following local injections of IGF-I that showed no significant improvement in the mechanical properties of treated tendons [18], indicating that local and systemic administration of IGF-I may act through different mechanisms. Furthermore, it is interesting that IGF-I alone positively influenced healing in tissues from both ambulatory and hindlimb unloaded animals, while combined GH and IGF-I only had a positive affect on tissues from HU animals. Since IGF-I increased tissue strength measures by ~60% in both ambulatory and hindlimb unloaded animals, it appears that IGF-I improves healing regardless of mechanical loading and that the affects of mechanical loading and IGF-I may be additive. The GH+IGF-I associated improvement observed only in hindlimb unloaded animals is more difficult to interpret. Nonetheless, this difference between ambulatory and HU animals after GH+IGF-I treatment may result from changes in the endocrine system due to unloading [55-57] or an inhibitory role of exogenously elevated GH on IGF-I function. Decreased levels of growth hormone, and changes in other endocrine factors, with unloading have been reported [55-60], and the addition of GH in unloaded rats may be simply re-establishing basal GH behavior in unloaded animals allowing the influence of increased IGF-I to proceed. In contrast, elevated levels of GH in ambulatory animals inhibit the positive affect of IGF-I, indicating that in fact elevated GH in ambulatory animals may not be permissive to elevated IGF-I function, but that improved healing from IGF-I addition is not dependent on exogenously elevated GH levels. Interestingly, addition of GH alone showed a trend of decreasing the mechanical properties of healing ligaments and hematomas in the healing site, further supporting the concept of a negative role for GH in ligament healing, even though combined supplementation of GH+IGF-I has been reported to increase serum IGF-I levels more than adding IGF-I alone [61,62]. Hence, the behavior reported herein is a complex phenomena superimposing the influence of mechanotransduction during tissue loading, local growth factor signaling, and endocrine hormone levels, yet implies a positive role for IGF-I and a negative role for GH in connective tissue healing through mechanisms that are, to date, not well understood.

Connective tissue atrophy and diminished levels of healing after disuse (e.g. spaceflight, hindlimb unloading, immobilization, etc.) is associated with reduced physical stimuli, however local growth factor signaling, and endocrine factors also play an important role. For instance, it is well established that microgravity or simulated microgravity disrupts pituitary GH function [58-60] and alters IGF-I expression and plasma concentration [60,63]. Interestingly, in this study the addition of GH alone, which can bind to cells via specific surface receptors activating numerous signaling pathways that direct changes in gene expression [64-67], did not offer any significant increase in mechanical properties. In contrast, the addition of IGF-I significantly improved the mechanical properties of tissues in treated animals, likely through structural improvements in the extracellular matrix as seen in Figures 4 and 5. In ambulatory animals treated with IGF-I and unloaded animals treated with IGF-I or GH+IGF-I, MCL matrix organization was greatly improved. This may be due to increased type-I collagen expression resulting in increased the matrix density, and altered cell behavior resulting in a more organized collagen matrix. Given the clear increases in matrix alignment, it appears probable that either collagen organization is initially improved during post-fibrillogenesis deposition or better organized during continued matrix remodeling, or both. Since collagen alignment is achieved before substantial tissue loading during fetal development [46,68], which is not reproduced during tissue repair in unloaded mature tissue [48], and it appears that matrix repair in adult tissue may be an imperfect reversion to processes seen in fetal development [46], addition of IGF-I may be stimulating signaling pathways similar to those seen during development. Moreover, one possible link in the molecular mechanism playing a role in increased matrix organization and collagen expression may be signals associated with IGF-I receptor signaling as indicated by increased IGFR levels in treated animals. However, IGFR activation was not examined in this study and multiple pathways associated with IGFR and matrix adhesion signaling (i.e. integrin signaling) are likely acting in concert to produce the profound improvements seen after IGF-I treatment. Hence, it is clear from these data that further work studying the systemic effects IGF-I need to be performed in order to better understand the mechanism of IGF-I administration on local tissue behavior.

It is known that soft tissue injuries do not fully recover even after long periods of healing [47] and stress reduction has a negative effect on healing in collagenous tissue, [48,52] which does not return to normal after re-establishing physiologic stress (i.e. remobilization) [69]. This pattern of healing is problematic since the injured joint often need immobilization, the growing aged population often experiences decreased activity levels or prolonged bed rest, and since prolonged space flight is becoming more feasible. Therefore, methods to improve tissue healing and counteract this negative decline during injury and/or disuse are increasing in need. The reported application of IGF-I clearly has a positive effect on tissue repair from a mechanical (functional) viewpoint, and therefore shows promise to improving normal tissue healing and to improving healing under normal or disuse conditions. Of particular note is the increase in force, stress, and elastic modulus in tissues from unloaded animals with IGF-I or GH+IGF-I, which become comparable to or surpass levels in ambulatory (Amb + Sal) animals. This improvement in tissue properties compares well with other methods to improve tissue repair, but may be more clinically feasible. For instance, application of PDGF-BB has also shown promising improvements in fibrous connective tissue healing. In two studies allowing unrestricted cage movement, Batten and co-workers [70] reported increases in maximal force up to 90% of controls, and Hildebrand and co-workers [71] reported increases in force of ~50% after PDGF-BB was administered locally immediately following injury. However, administering PDGF-BB 48 hrs post-injury resulted in decreased force values [70], while administering IGF-I post-injury herein improved tissue healing. Therefore, the results presented in this paper showing an increase in maximal force of ~60% in ambulatory animals after 3 weeks of systemic IGF-I compares favorably to the levels of improvement previously reported in the literature but treatment can begin post-injury. Yet, although short term systemic application of IGF-I or GH+IGF-I provide compelling evidence for improved healing in a collagenous matrix, potential side effects of altering GH and IGF-I levels for long periods of time have not yet been fully explored in conjunction with these data and therefore further study is required before long term use is warranted in humans.

## Conclusion

In conclusion, results support our hypothesis that systemic administration of IGF-I improves healing in collagenous extracellular matrices. Growth hormone alone did not result in any significant improvement contrary to our hypothesis, while GH + IGF-I produced remarkable improvement in hindlimb unloaded animals. Interestingly, addition of IGF-I or GH + IGF-I in HU animals resulted in recovery of strength measures to a level equal to ambulatory controls, indicating that in fact IGF-I may be a plausible therapy for overcoming reduced tissue healing due to disuse from bed rest, immobilization, or microgravity. Additionally, although supplementation with IGF-I in ambulatory animals did not result in full recovery of the mechanical properties at 3 weeks, treatment resulted in an ~60% increase in tissue strength demonstrating the potential for IGF-I to improve tissue healing. These changes in tissue healing with tissue loading or IGF-I supplementation raise important questions regarding the essential role of mechanical stress for collagen matrix organization in connective tissues and the mechanisms by which systemic IGF-I or GH+IGF-I lead to improved tissue healing.

## Methods

### Animal model and study design

This study was approved by the institutional animal use and care committee and meets N.I.H. guidelines for animal welfare. Seventy-two male Sprague-Dawley rats (248 ± 6 grams) were used as an animal model. These animals were randomly divided into nine groups each containing eight rats: **1) **sham control surgery - ambulatory + saline (Sham), **2) **healing - ambulatory + saline (Amb + Sal), **3) **healing - hindlimb unloaded + saline (HU + Sal), **4) **healing - ambulatory + GH (Amb + GH), **5) **healing - hindlimb unloaded + GH (HU + GH), **6) **healing - Ambulatory + IGF-I (Amb + IGF), **7) **healing - hindlimb unloaded + IGF-I (HU + IGF), **8) **healing - ambulatory + GH + IGF-I (Amb + GH + IGF), **9) **healing - hindlimb unloaded + GH + IGF-I (HU + GH + IGF). Each rat in the ambulatory healing and hindlimb suspension (unloaded) groups underwent aseptic surgery to disrupt both knee MCLs. Rats were anesthetized with an isofluorane anesthetic administered by facemask using a non-rebreathing delivery system. The incision area was clipped and prepared for surgery. A skin incision approximately 8 mm long was made on the medial side of the stifle and fascia was incised to expose the MCL. The MCL was exposed and transected at the joint line, after which the muscles and skin were closed with suture. Sham control animals were subjected to identical surgical procedures without MCL disruption. All animals were given an analgesic (Tylenol^®^/codeine) in their water for 72 hours post-surgery. Animals in the sham control and ambulatory healing groups were allowed unrestricted cage movement. Hindlimb unloaded healing rats were subjected to hindlimb unloading (24 hours after surgery), which induced substantial stress reduction by eliminating ground reaction forces, using the noninvasive tail suspension protocol of the NASA-Ames center [72]. Only the hindlimbs were suspended (unloaded) and the animals were allowed to move freely within the cages using the forelimbs while being restricted from kicking the sides of the cages. For GH and IGF-I delivery, recombinant human GH or IGF-I (Genentech, South San Francisco, CA) was dissolved in sterile saline at a concentration of 0.25 mg/mL. Rats received two 0.5 mL injections daily (0830 and 1530 hours) of GH, IGF-I, or GH+IGF-I subcutaneously in the nape of the neck. The dosage of GH and IGF-I were based upon previous studies in rats [73-75] based on initial body weight and was not adjusted during the course of the study. Combined GH and IGF-I were administered since previous reports have shown that increased levels of serum IGF-I and IGF-I binding proteins are obtained after co-injection of GH and IGF-I when compared to either GH or IGF-I alone [61,62]. All animals were housed at 24°C under a 12 hr light and 12 hr dark cycle, fed Purina rat chow, and watered *ad libitum*. Rats were checked twice daily for overall health, skin incision healing, food and water consumption, and the condition of their tails (the harness should prevent slippage without restricting circulation). After 3 weeks the animals were euthanized. Immediately after death, animal hindlimbs were flash frozen and stored at -80°C until testing. Of the sixteen ligaments per group, six ligaments were used for mechanical testing, six for histology and immunohistochemistry, and four for Western blotting.

### Biomechanical testing

On the day of testing, hindlimbs were thawed at room temperature from -80°C. It has been shown that postmortem storage by freezing does not change the biomechanical properties of ligament [76]. Tissue harvest and testing were performed using methods similar to those previously described [48,77]. Extraneous tissue was carefully dissected away to expose each MCL and the femur-MCL-tibia complex was removed. Ligaments were kept moist in PBS at 25°C to prevent dehydration (pH = 7.4), and cross sectional area measured optically. While remaining hydrated the femur-MCL-tibia complex was placed into a custom designed tissue bath system with special structures to hold the femoral and tibial bone sections of the sample along the longitudinal axis of the MCL where all fibers appear to load simultaneously (~70° flexion). For tissue strain measurement, optical markers (impregnated carbon grease) were placed onto the ligament tissue near the insertion sites and the tissue bath containing the MCL samples were inserted into our custom testing machine. A small preload of 0.1 N was applied in order to obtain a uniform start (zero) point. The ligaments were preconditioned (~1% strain for 10 cycles) to allow the tissues to settle into the grips of the testing bath and then they were pulled to failure in displacement control at 10 %/s. Tissue deformation was examined and recorded until post-failure. Digital images of each test were analyzed to assess the structural failure location. For the case of a potential tibial avulsion the distal end of the ruptured tissue was examined for bone.

Tissue displacement was obtained using video dimensional analysis (resolution = 10 μm). The change in distance between optical markers was calculated by analyzing digital frames from the test with N.I.H. Image using a custom macro to calculate the change in x-y coordinate center (centroid coordinates) of each marker. Force (resolution 0.005 N) was obtained, displayed on the video screen and synchronized with displacement. From displacement data, engineering strain was calculated where L_0 _is the initial length of the tissue at preload (0.1 N) and L is the current distance between strain markers:

ε=L−L0L0=λ−1.     (1)
 MathType@MTEF@5@5@+=feaafiart1ev1aaatCvAUfKttLearuWrP9MDH5MBPbIqV92AaeXatLxBI9gBaebbnrfifHhDYfgasaacH8akY=wiFfYdH8Gipec8Eeeu0xXdbba9frFj0=OqFfea0dXdd9vqai=hGuQ8kuc9pgc9s8qqaq=dirpe0xb9q8qiLsFr0=vr0=vr0dc8meaabaqaciaacaGaaeqabaqabeGadaaakeaaiiGacqWF1oqzcqGH9aqpdaWcaaqaaiabdYeamjabgkHiTiabdYeamnaaBaaaleaacqaIWaamaeqaaaGcbaGaemitaW0aaSbaaSqaaiabicdaWaqabaaaaOGaeyypa0Jae83UdWMaeyOeI0IaeGymaeJaeiOla4IaaCzcaiaaxMaadaqadaqaaiabigdaXaGaayjkaiaawMcaaaaa@3F3B@

The stretch ratio is represented by λ and is the current length divided by the original length. Lagrangian stress was calculated as the current force (F) divided by the initial undeformed area (A_0_):

σ=FA0     (2)
 MathType@MTEF@5@5@+=feaafiart1ev1aaatCvAUfKttLearuWrP9MDH5MBPbIqV92AaeXatLxBI9gBaebbnrfifHhDYfgasaacH8akY=wiFfYdH8Gipec8Eeeu0xXdbba9frFj0=OqFfea0dXdd9vqai=hGuQ8kuc9pgc9s8qqaq=dirpe0xb9q8qiLsFr0=vr0=vr0dc8meaabaqaciaacaGaaeqabaqabeGadaaakeaaiiGacqWFdpWCcqGH9aqpdaWcaaqaaiabbAeagbqaaiabbgeabnaaBaaaleaacqaIWaamaeqaaaaakiaaxMaacaWLjaWaaeWaaeaacqaIYaGmaiaawIcacaGLPaaaaaa@368B@

Stress versus stretch-ratio curves were created and fit with the microstructural model presented by Hurschler and co-workers [78] to obtain the elastic modulus. Biomechanical parameters for comparison are ultimate force, ultimate stress, strain at failure, and elastic modulus (determined from the microstructural model).

### Histology and immunohistochemistry

Immediately following tissue harvest, samples for histology were fixed in 10% formalin. After standard histology procedures, sections underwent hematoxylin and eosin (H&E) staining. Slides were coverslipped and viewed with light microscopy. For immunohistochemistry, fixed ligaments were flash frozen in Optimal Cutting Temperature Media (OCT) at -70°C. Cryosections (6 μm) of each MCL were mounted onto lysine coated slides for staining. Mounted specimens were washed in a solution of PBS and 0.1% Tween-20 (PBST) between all incubation steps. Endogenous peroxidase activity was blocked by incubating slides in 3% hydrogen peroxide for 10 minutes, followed by blocking with 5% goat serum for 30 minutes at room temperature in a moist chamber. Tissue sections were then incubated for one hour at room temperature in a 1:1 dilution of SP1.D8 (University of Iowa Developmental Studies Hybridoma Bank), a primary antibody against the amino-terminal of type I procollagen. Sections were then incubated in rat adsorbed secondary antibody (Innovex Biosciences, Parsippany, New Jersey) for 20 minutes at room temperature, followed by incubation in horseradish peroxidase (HRP) labeled strepavidin (Innovex Biosciences, Parsippany, New Jersey) for 20 minutes at room temperature. Slides were stained with DAB (3,3'-diaminobenzidine; Innovex Biosciences, Parsippany, New Jersey) for 5 minutes at room temperature, and then washed in distilled water (twice) for 5 minutes. No counterstaining was performed and no primary antibody and a species appropriate IgG antibody served as negative controls. All slides were dehydrated in graded ethanol solutions, and then cleared in xylenes prior to cover-slipping. Light microscopy was used to examine each specimen and digital images of each MCL specimen were taken at 100× magnification. The area of staining was measured using ImageJ software after establishing a constant threshold across images.

### Multiphoton laser scanning microscopy

Multiphoton Laser Scanning Microscopy (MPLSM) was performed on hematoxylin and eosin stained slides using an optical workstation [79,80] to better examine collagen organization in tissue sections. Since, MPLSM of formalin fixed paraffin embedded sections produces images of collagen structure and organization that far exceed the detail provided with standard histology under brightfield illumination, MPLSM was employed to detect potential differences in collagen matrix organization that have been previously reported in healing ligaments from ambulatory and hindlimb unloaded animals using scanning electron microscopy [48]. The excitation source was a Ti:sapphire laser (Spectra-Physics-Millennium/Tsunami, Mountain View, CA) producing around 100fs pulse widths and tuned to 890 nm. The beam was focused onto the sample with a Nikon 40X Plan Fluor oil-immersion lens (N.A. = 1.4).

### Western blot analysis

Ligaments were finely minced and boiled in 3X Laemmli buffer for 10 minutes. Following centrifugation to remove tissue debri, protein was separated by SDS-PAGE on 7.5% gel, and electro-transferred to PVDF membrane (Millipore, Billerica, MA). Membranes were blocked with TBST (10 mM Tris, 150 mM NaCL, 0.3% Tween-20) plus 5% Blotto (Santa Cruz Biotechnology, Santa Cruz, CA), followed by incubation with either rabbit anti-collagen type-I (Biotrend, Germany) or mouse anti-collagen type-III (Abcam; FH-7A) for 30 minutes at 37°C or anti-IGF-I receptor antibody (Cell Signaling Technology, Danvers, MA) overnight at 4°C. The membrane was then incubated in secondary HRP-conjugated donkey anti-rabbit or anti-mouse antibody (Jackson ImmunoResearch Lab; 0.8 μg/mL) and visualized using the ECL Plus detection reagent (Amersham, Piscataway, NJ). For analysis of the ratio of collagen I to III the membranes were probed for type III collagen then stripped and re-developed to confirm the absence of residual signal, and then reprobed for type I collagen. IGF-I receptor levels were normalized to levels of GAPDH (anti-GAPDH; Santa Cruz Biotechnology, Santa Cruz, CA). Following automated development of a number of exposures to confirm linearity, densitometry analysis was performed with ImageJ software. The values for each group were normalized to the Sham group to allow comparison of data across multiple Western blot experiments. Specificity of the collagen antibodies was confirmed by blotting purified type I and III collagen (BD Biosciences) and a panel tissues known to be composed of types I and III collagen. Cross-reactivity of the antibodies for other collagens was negligible (data not shown). The anti-collagen type I antibody did not cross-react with type III collagen consistent with the manufacturers report that the antibody has minimal cross reactivity to types II, III, IV, V, and VI collagen. The anti-collagen type III antibody did not substantially cross-react with type I collagen consistent with the manufacturers data indicating that the antibody does recognize collagen types I, II, IV, V, VI and X.

### Statistical analysis

Due to the non-factorial nature of our experimental design, statistical analysis was performed using a one-way analysis of variance (ANOVA) followed by pair wise comparisons. The level of significance was set to 0.05 and analysis was performed with SAS PROC MIXED (SAS Institute, Inc., Cary, NC). Fisher's protected least significant difference test was used for post-hoc multicomparisons. For analyzing failure location data, the non-parametric Kruskal-Wallis test was performed.

## Abbreviations

Extracellular matrix (ECM), Growth hormone (GH), Glyceraldehyde-3-phosphate dehydrogenase (GAPDH), Hindlimb Unloaded (HU), Insulin-like growth factor I (IGF-I), Insulin-like growth factor receptor (IGFR), Medial collateral ligament (MCL), Multiphoton Laser Scanning Microscopy (MPLSM), Platelet derived growth factor (PDGF); *Group Abbreviations*: sham control surgery - ambulatory + saline (Sham), healing - ambulatory + saline (Amb + Sal), healing - hindlimb unloaded + saline (HU + Sal), healing - ambulatory + GH (Amb + GH), healing - hindlimb unloaded + GH (HU + GH), healing - Ambulatory + IGF-I (Amb + IGF), healing - hindlimb unloaded + IGF-I (HU + IGF), healing - ambulatory + GH + IGF-I (Amb + GH + IGF), healing - hindlimb unloaded + GH + IGF-I (HU + GH + IGF).

## Authors' contributions

PPP conducted and analyzed all biomechanical testing and multiphoton microscopy experiments, performed analysis of histology, assisted in immunohistochemistry analysis and western blotting with AAO, and prepared the manuscript and figures. AAO additionally contributed with manuscript preparation. KWG performed immunohistochemistry experiments. REG managed animal treatments and tissue harvest (with DAM). DAM, REG, and ACV participated in the study design (with PPP and RV), performed and/or supervised animal surgeries, and coordinated IGF-I and GH treatment. RV oversaw all aspects of the study and assisted in manuscript preparation. All authors assisted in critical editing the manuscript.
